# *Psophocarpus tetragonolobus*: An Underused Species with Multiple Potential Uses

**DOI:** 10.3390/plants9121730

**Published:** 2020-12-08

**Authors:** Hussein Bassal, Othmane Merah, Aqeel M. Ali, Akram Hijazi, Fawaz El Omar

**Affiliations:** 1 Doctoral School of Science and Technology, Research Platform for Environmental Science (PRASE), Lebanese University, Beirut, Lebanon; hussein.bassal@ul.edu.lb; 2 Laboratory of Cancer Biology and Molecular Immunology, Faculty of Sciences, Lebanese University, Hadath-Beirut, Beirut, Lebanon; 3 Laboratoire de Chimie Agro-industrielle, LCA, Université de Toulouse, INRA, 31030 Toulouse, France; 4 Département Génie Biologique, Université Paul Sabatier, IUT A, 32000 Auch, France; 5 Department of Biology, College of Science, Al Mustansiriya University, Baghdad, Iraq; aqeelm.ali@uomustansiriyh.edu.iq; 6 Doctoral School of Science and Technology, Lebanese University, EDST, Hadath, Beirut, Lebanon; fomar@ul.edu.lb

**Keywords:** winged bean, ethnomedicinal uses, therapeutic activities, anti-inflammatory effect, anti-proliferative activity, nutrients

## Abstract

Natural products, particularly those extracted from plants, have been used as therapy for different diseases for thousands of years. The first written records on the plants used in natural medicine, referred to as “medicinal plants”, go back to about 2600 BC. A thorough and complete understanding of medicinal plants encompasses a multiplex of overlapping and integrated sciences such as botany, pharmacognosy, chemistry, enzymology and genetics. *Psophocarpus tetragonolobus*, a member of *Fabaceae* family also called winged bean, is a perennial herbaceous plant characterized by its tuberous roots and its winged pod twinning and a perennial legume rich in proteins, oils, vitamins and carbohydrates. Besides nutrients, winged bean also contains bioactive compounds that have therapeutic activities like anti-oxidant, anti-inflammatory, antinociceptive, antibacterial, antifungal, antiproliferative and cytotoxic activity, a few of which already been reported. This plant can also be used as a medicinal plant for future benefits. With this concept in mind, the present review is designed to shed the light on the interests in the various phytochemicals and pharmacological pharmacognostical aspects of *Psophocarpus tetragonolobus*.

## 1. Introduction

Over time and since the dawn of societies, humans have learned to probe and categorize plant materials to meet the essentials of life. Herbs and their extracts were used for their healing powers. The medicinal plants sector has undergone a remarkable evolution, especially during the last decade. The global market is moving more and more towards products of natural origin. Plants that are used in natural medicine were referred to as “medicinal plants” [1].

During the 19th century, several alkaloids isolated from unique plant species were used as medicinal drugs such as atropine from “*Atropa belladonna*” *(Solanaceae*), saclin from “*Salix*” species (*Salicaceae*) and morphine and codeine from “*Papaver somniferum*” (*Papaveraceae*). Subsequently, the bioactive secondary metabolites derived from plants were widely used as drugs in their original and modified forms [2]. A direct relationship between the use of herbal plants and modern drugs isolated from these plants has been established, where 88 compounds isolated from seventy-seven medicinal plants were then introduced into the current treatments [3]. Nowadays, most of the world’s population still depends on plants for their primary health care according to the World’s Health Organization (WHO). In this review, we will discuss the chemical constituents, biological properties and benefits of *Psophocarpus tetragonolobus.*

## 2. *Psophocarpus tetragonolobus:* Botany, Genetic and Cultivation

*Psophocarpus tetragonolobus* is also called *Lotus tetragonolobus*, asparagus pea, goa bean, four-angled bean and winged bean [4]. Winged bean is a tropical leguminous plant that is listed as one of the underutilized legumes and it is an underexploited food source for the tropics. *P. tetragonolobus* is known as “poor man’s food” since the leaves, flowers, roots, and pods are eaten raw or cooked. Apart from being an edible plant, the fruits are reported to have anti-inflammatory, antioxidant, and anti-nociceptive activities [4].

### 2.1. Plant Taxonomy

The winged bean is one of six species of the genus *Psophocarpus* and the family *Leguminosae (Fabaceae)* as shown in Table 1. *Psophocarpus tetragonolobus* is a twining, perennial herb that’s characterized by its tuberous roots and its winged pods [5].

### 2.2. Origin and Distribution

Winged bean, a domestic plant, is distributed in South East Asia, in the countries situated between India and Papua New Guinea, along with some African countries. The relation between *Psophocarpus tetragonolobus* and other *Psophocarpus* species is mainly identified depending on morphology [5,6]. However, comparing the genetic makeup of these plants will be a more reliable approach that can help to identify the wild ancestor of this plant. Till now, different theories are suggested concerning the origin of this plant. Southeastern Asia was suggested as an origin because the plant was cultivated in this area for a long time. However, the progenitor was not known and it was assumed to be extinct. *Papua New Guinea* is another possible origin due to the large repertoire of genetic varieties of plants in this country. Winged bean was also found to be similar to some African plants in terms of morphology, cytology and pathology [7].

### 2.3. Morphology

**Figure 1:**
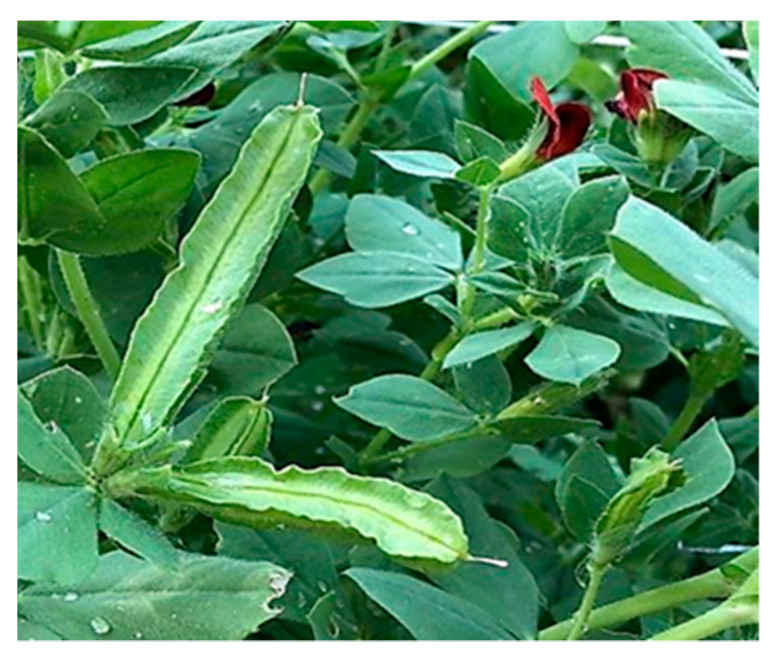
A *Psophocarpus tetragonolobus* plant.

Winged bean is a climbing twinning plant (climb up to 3–5 m). It has green trifoliate leaves made up from three leaflets of ovate to deltoid shape. It produces 2.5 to 3.5 cm wide flowers with colors ranging from purple, white and blue, blue to red. The pollen grains are spheroidal with a polar axis that measures between 42.3 and 51.6 cm and an equatorial axis that measure between 43.4 and 49.9 cm. Its roots are tuberous; a tuber ranges in size between 8 and 12 cm in length and 2 to 4 cm in width. It produces elongated pods (the fruit) with four corners (Figure 1) and at each one the pod bears a wing, hence the name of winged bean. Each pod ranges from 15 to 30 cm in length, and it is about 3 cm in width [8].

At the cellular level, a study of seven plants of winged bean from Okianawa (Japan) revealed that its cells have thick cell walls with plasmodesmata. Cotyledonary cells analysis was done after flowering. At day 30, starch granules in amyloplasts are observed. Also, the cells contained tubular rough endoplasmic reticulum and vacuoles containing dense flocculent material. At day 45, lipid and protein bodies appear [9].

### 2.4. Diversity and Molecular Characterization

Understanding the genetic diversity of a plant species is important for producing genetic improvement for better characteristics and benefits. Moreover, analyzing the variability in traits between plants of different origins, and comparing those traits to the genome and transcriptome may help in molecular breeding to produce plats of winged bean with desirable agronomic traits [10].

The genetic resources for winged bean are not rich because its ancestor is not yet identified. Nevertheless, the comparison of its genome with other legumes that were fully sequenced can be interesting. Those legumes include: *Glycine max, Cicer arietinum, Lotus japonicas, Cajanus cajan, Phaseolus vulgaris and Medicago truncatula* [10]. More studies are now performed also for sequencing of the genome of *Lupinus angustifolius, Trifolium pratense, Pisum sativum and Arachis hypogea* [11]. Nowadays, in conjugation to the already published sequencing data, the available inexpensive genomic techniques open an avenue for investigating the genomic makeup of the winged bean. A study on 24 accessions of winged bean was done and identified that ISSR markers were more efficient than RADP markers. Consequently, Chen et al. studied ISSR markers in 45 accessions to understand genetic variability, and the results of genetic distance and genetic identity between the accessions of winged bean showed a closed relationship and narrow genetic background. [12]. On the other hand, Vatanparast et al. strengthened the genetic resources for *Psophocarpus tetragonolobus* by producing a *de novo* transcriptome assembly and annotation of two Sri Lankan accessions (denoted herein as CPP34 [PI 491423] and CPP37 [PI 639033]), developing simple sequence repeat (SSR) markers, and identifying single nucleotide polymorphisms (SNPs) between geographically separated genotypes [13]. Another transcriptomic study was done on seedling from winged bean (Ibadan Local-1) and identified around 1900 microsatellites and around 1800 conserved orthologous loci [14], also Wong et al. sequenced transcriptomes of plants from Malaysia and found 9682 SSR markers, among which 18 were validated for nine accessions [15], these sets of microsatellite markers could be used to contribute to genetic linkage maps in winged bean, with the integration of single nucleotide polymorphisms (SNPs) markers for higher density.

Comparison of gene expression between winged bean plants contrasted for their tannin production revealed more than 1000 differentially expressed genes. This result allowed developing and cultivating cultivars with low tannin content [16]. Furthermore, twenty-two mutations were found with desirable outcomes in terms of morphology and chlorophyll content. However, these mutants were sterile. A gene that has anti-fungal effects (class III chitinase) was shown to be expressed in the roots of winged bean plants [17].

Unfortunately, this species received less attention that other economically important Fabaceae species (soybean, peanut for example) to develop breeding programs to improve plant resistance against major diseases or pests.

### 2.5. Cultivation

Winged bean is usually cultured in tropical countries. *Psophocarpus tetragonolobus* can live at a wide range of altitudes (from 0 to 2000 m) and in different types of soils, even those with a low amount of nutrients [18]. This is due to the presence of symbiotic bacteria, mainly *Rhizobium* strains, in nodules at the roots, which can concentrate nitrogen transported to the plant as allantoic acid and allantoins [19].

Winged bean cannot withstand soils with pH < 5.5 or water-soaked [20]. Before planting, seeds must be soaked in water, and fungicides are commonly applied. The distance between seeds is around 50 cm. It is self-pollinating plant, but cross-pollination can also occur (7.6%). In the first 6 weeks, *Psophocarpus tetragonolobus* grows slowly, and after 10–12 weeks of planting, the fruit can be harvested. With respect to pods and seeds, it produces the highest yield in the first year of plantation, so it is planted annually. However, this plant is long living, and it was shown that the tuber yield is much better in the second-year plantation 369 to 392 g for each plant compared to 80 to 230 g in the first year [21].

Winged bean is affected by different diseases caused by viruses, pests, fungi and nematodes. With respect to pests, winged bean is affected by a repertoire of species: *Orthoptera, Coleoptera, Thysanoptera, Diptera, Hemiptera and Lepidoptera*. The leaves can be damaged by the winged bean blotch miner (*Leucoptera sophocarpella*), the flowers by the bean pod borer (*Maruca testulalis*), cotton boll worm (*Helicover armigera*), *Mylabris afzelii* and *Mylabris pustulata*. Bean pod borer and cotton bollworm infect also the fruit [22].

Some pests can infect the leaves, the flowers and the shoots. These are: the black bean aphid (*Aphis craccivora*), the lady bird (*Henosepilachna signatipennis*), the bean fly (*Ophiomyia phaseoli*), the pea blue butterfly (*Lampides boeticus*), southern green stink bug (*Nezara viridula*), *Tetranychus urticae*, *Podalia spp*. and *Polyphagotarsonemus* [23]. In Sri Lanka, this plant was affected by *Riptortus pedestris*, *Hypolixus truncatulus, Myllocerus undatus, Myllocerus curvicornis, Luperomorpha discoidea, Parasa lepida, Syntomis passalis, Euproctis scintillans, Dysdercus olivaceus, Pagriasignata, Helopeltis species, Leptocentrus, Laius, Euops, Zoriada, Brachyplatys and Cletus* [24]. Many nematodes were shown to affect winged bean plant, among which 70% of the loss in tubers accounted to infections by *Meloidogyne arenaria*, *M. incognita* and *M. javanica*. In Ivory Coast, the roots and tubers of the winged bean were affected by *Meloidogyne spp* [23].

Winged bean is affected by five main fungal diseases; Choanephora blight (caused by *Choanephora cucurbitarum)*, collar rot disease (caused by *Fusarium semitectum*, *F. moniliforme, F. equiseti, Rhizoctonia solani and Macrophomina phaseolina)*, powdery mildew (caused by *Erysiphe cichoracearum*), dark leaf spot disease (caused by *Pseudocercosa psophocarpi)*, and finally, the false rust disease, one of the most common diseases, caused by *Synchytrium psophocarpi*. This disease is characterized by orange pustules that appear on leaves, pods and stems. In a study on *Synchytrium psophocarpi* infected winged bean plants from Malaysia, it was found that the sporangia were 20.69 µm in size and their shape was spherical to ovoid, the spores were 2.02 µm in diameter with flagella of length equal to 10.75 µm. Three more fungal diseases also appeared in Sri Lanka; pulvinus rot disease (caused by *Fusarium pallidoroseum*), leaf scorch disease (caused by *Phomasogina*) and pod rot disease *(Botryodiploidea theobromae*) [25].

Viruses can also infect *Psophocarpus tetragonolobus*: Ecrotic mosaic virus infects 9% of young plants and ring spot mosaic virus is known to be responsible of 10 to 20% of yield loss. Leaf curl disease, found in Ivory Coast, and winged bean endornavirus 1 is a new virus that appeared lately [22,25].

## 3. Food Utilization

### 3.1. Consumption and Processing

Winged bean is consumed mainly in South Africa, Southern Asia, India and Malaysia [26]. All the parts of winged bean plants can be eaten, including the pods (raw or pickled, consumed largely in India [6]), flowers (used in salad and to color dishes [8]), leaves (prepared and eaten similar to spinach), roots (tubers. raw or cooked especially in Burma, Ghana, Papua New Guinea, Thailand and Indonesia) and seeds. The seeds are processed and consumed in diverse ways: Unripe seeds are eaten in soups [26,27]; mature seeds can be eaten roasted, or dried and grounded to produce flour. Furthermore, oil can be extracted from the seeds and used in cooking or frying food.

### 3.2. Nutrient Composition

Winged bean plant is appreciated for the high nutrients composition, especially with respect to proteins, vitamins and minerals; the tubers are starchy with high percentage of proteins 17 to 20% (by weight) compared to other vegetables, the leaves and flowers contain from 5 to 15% proteins (by weight), the seeds are highly nutritious with 32 to 37% proteins, which is similar to the amount of proteins found in soy beans and higher than that found in other beans [26]. The seeds also contain 23 to 40% carbohydrates [8,28,29], vitamins like vitamin B_1_, B_2_, B_3_, B_6_, B_9_ and in vitamins C, A and E. The mature seeds contain 14 to 25% fats by weight, of which 94% are in free form, whereas the rest are complexed with carbohydrates and proteins [29].

Winged bean is rich in minerals including calcium, iron, phosphorous, potassium, sulfur, sodium, magnesium, zinc, manganese, boron, barium, copper, chromium and strontium [28,30,31]. *Psophocarpus tetragonolobus* contains 54 to 75% of unsaturated fatty acids, of which 38.6% are mono-unsaturated and 36.9% are polyunsaturated [29,32], and no *trans* fatty acids are found. Winged bean oil contains 30 to 40% saturated fatty acids which represent more than the content observed in soybean [33]. Oleic and linoleic acids represent nearly 50% of unsaturated fatty acids. Unsaponifiable lipids are mainly represented by β-sitosterol (66.4%) and stigmasterol (25.1%) [29]. Moreover, winged bean oil was found to be better than soybean oil, because of its high oxidative and high thermal stabilities [34].

### 3.3. Anti-Nutrient Composition

Winged bean contains many anti-nutritional factors: trypsin inhibitors, chymotrypsin inhibitors (WCI), hemagglutinins, amylase inhibitors, phytates, phytic acid, flatulence factors, hydrogen cyanide, saponins, tannins and other phenolic compounds [35,36]. Table 2 shows the main phytochemicals of *Psophocarpus tetragonolubs* responsible of its anti-oxidant and anti-inflammatory activities.

Trypsin inhibitors are mainly present in seeds where its activity is found between 11,300 and 74,700 IU/g of seeds which varies according to the cultivar [19,30]. Winged bean chymotrypsin inhibitors (WCI) are mainly present in tubers and a seed, its activity exceeds that of trypsin inhibitor, with 1 g of beans able to inhibit approximately 30 to 48 mg of chymotrypsin [35]. WCI are encoded by many genes and they can be distinguished as Bowman-brick-type inhibitors (three are identified till now) and Kunitz inhibitors KI (9 are identified till now) [35,37]. Three new KI genes were isolated lately: WCI-3b, WCI-2 and WCI-5. WCI-5, expressed only in the seeds, was confirmed to have high proteinase inhibitor activity and inhibit the growth of larvae of *Helicoverpa armigera*, protecting the plant against this pest [38,39].

*Psophocarpin* B1, B2 and B3, winged bean proteins, were shown to have similarities with lectins of soybean. Hemagglutinin activity was detected in winged bean accounting to 77 to 154 hemagglutinin unit/g of winged bean [40]. Till now, two hemagglutinins were identified in winged bean (WBA-I and WBA-II), which were able to agglutinate erythrocytes belonging to different blood groups [41]. However, hemagglutinin along with trypsin and chymotrypsin inhibitors are destroyed when heated, therefore only raw seeds will contain that amount of these anti-nutritive factors. Autoclaving the flour obtained from seeds for brief time led to a decrease in the activity of the three of them; from 30 to 40% decrease in activity in case of trypsin inhibitors, 15% decrease in case of chymotrypsin inhibitor and the great decrease was for hemagglutinins which ranged between 75 and 96%. After autoclaving the flour for 30 min, the activities of all the three components was abolished. However, a high percentage of protein became insoluble [31].

Tannins and other phenolic compounds are also present in the seeds in quantities ranging from 0.03 to 7.5 mg/g of beans, lower than the amount of tannins in other beans [42]. They can inhibit enzymes non-specifically and precipitate with proteins and thus lower the amount of available protein in the food. Phytates are other anti-nutritional factors that are present in seeds in amounts between 6.1 and 7.5 mg/g of seeds, which is similar to the amount present in soy bean. However, these amounts are not very high, so it is not harmful [36]. Another component is present in the wax of mature leaves which is alkanes. Many *n*-alkanes are present in this wax ranging from *n*-C_16_ to *n*-C_20_ and from *n*-C_22_ to *n*-C_35_. Among these alkanes, *n*-C_29_ and *n*-C_31_ were the most abundant whereas *n*-C_20_ and *n*-C_26_ were the least abundant [43].

## 4. Therapeutic Potential of *Psophocarpus tetragonolobus*

Winged bean was used since a long time as a medicinal plant in different countries: its fruits and roots were used as medicines that increase strength, and as treatment of ulcers in New Guinea [49]. Moreover, its leaves were used as treatment of small pox and its tubers were used in the treatment of vertigo in Malaya, both for external use. Nowadays, different studies were done to investigate the anti-oxidant, antimicrobial, anti-inflammatory and antiproliferative activities of different extracts from this plant [50,51].

### 4.1. Anti-Oxidant Activity of Psophocarpus tetragonolobus

Winged bean is a source of many known antioxidants like vitamin C, and it is also rich in polyphenols and flavonoids. Phenolic compounds are very important as antioxidants. Their antioxidant effects anticarcinogenic, anti-oxidant, anti-inflammatory, antitumoral, antimicrobial, antimutagenic, anti-aggregate, anti-ischaemic and anti-allergic. Phenolics also alleviate cardiovascular diseases [52,53]. Phenolic compounds are responsible for the antioxidant activity of fruits as a result of their redox properties that allow them to act as reducing agents, hydrogen donors, singlet oxygen quenchers and metal chelators [54]. Flavonoids are secondary metabolites widely found in fruits, vegetables and legumes. They have been linked with several biological activities which include antioxidant, anti-inflammatory, antiviral and anticancer effects [55]. Several studies have showed that the consumption of flavonoid-rich foods protect against diseases associated with oxidative stress [56,57]. In the seeds, the phenolic content ranged between 0.8 and 0.9 mg gallic acid equivalent/g and the total flavonoids ranged between 0.7 and 1.2 mg quercetin equivalent/g, and the total antioxidant capacity was between 1.3 and 1.8 mg AAE/g. Thus, they can serve as sources of health-promoting nutrients and phytochemicals for human and animals. Different studies examined various extracts and fractions of winged bean for their antioxidant activities, using different techniques [58].

A 75% methanol extracts and its ethyl acetate and chloroform fractions, *n*-butanol, petroleum ether, and the methanolic plant extracts all showed an antioxidant activity. By ferric reducing antioxidant power (FRAP() and ferric thiocyanate (FTC) techniques, the ethyl acetate fraction of the methanolic extracts had the highest antioxidant activity and the highest phenolic content (1.7 mg GAE/g) compared to the chloroform fraction and the methanol extract itself. However, by the 2,2′-azinobis-(3-ethylbenzothiazoline-6-sulphonic acid (ABTS) assay, the chloroform fraction had the highest antioxidant activity. *n*-Butanol and petroleum ether extracts were proved to have anti-oxidant activity using ABTS and FRAP assays, and the phenolic content was shown to be correlated with the anti-oxidant activity.

Total phenolic contents are influenced by the concentration of extract. Among all the fractions, the ethyl acetate fractions exhibited the highest total phenolic content comparing to methanol and chloroform fraction. It was suggested that the antioxidant activity of *P. tetragonolobus* was highest in chloroform extract as compared to methanol and ethyl acetate [50].

### 4.2. Anti-Bacterial and Anti-Fungal Properties of Psophocarpus tetragonolobus

The anti-microbial activity of the winged bean was manifested as both antibacterial and anti-fungal effects. A methanol extract from the leaves or from the roots was shown to kill and inhibit the growth of both *Pseudomonas aeruginosa* (a bacterium species) and *Candida albicans* (a species of fungus), respectively. This was proved using the disk diffusion and the broth dilution assays. In the case of *Pseudomonas aeruginosa*, the minimum inhibitory concentration (MIC) was 2.6 mg/mL, whereas in the case of *Candida albicans*, the MIC was 3.1 mg/mL. In both cases, after 36 h of incubation, there was a morphological change of the microbe as examined by a scanning electron microscope. The extract was tested in vivo on rats to study toxicity, in the dose of 2 g/kg, and no rats were killed. Different fractions of extracts from tubers, leaves, stems and pods were studied by disk diffusion assay for their anti-microbial activity. The fractions used were chloroform fraction, ethyl acetate fraction and ethanol fraction. All were effective against all the tested microbes (11 bacteria, four molds and four yeasts), with the pod having the highest activity and the leaves having the lowest. All the fractions were effective with the ethanol fraction having the highest activity, whereas chloroform fraction had the lowest activity [16,59].

### 4.3. Anti-Proliferative Activity of Psophocarpus tetragonolobus

In a study on human colon cancer cell line (HT-29), a methanol extract was shown to have strong antiproliferative activity using the sulforhodamine B assay. However, the *n*-butanol fraction had even higher activity [60]. On the other hand, *Helicobacter pylori* are a bacterial species that targets the gastric epithelium and may cause gastric cancer. The infection is dependent on the binding of bacteria to gastric epithelial cells, and this binding requires MUC1 and MUC5AC, which are mucins found on the gastric cells. The high level of proteins in the *P. tetragonolobus*, namely, lectins, is used as a diagnostic tool because it binds certain blood cells and specialized transport cells [61]. Lectins from winged bean were able to decrease the expression of these mucins and two other antigens (Lewis b and H type 1) in gastric cells as shown by ELISA assay [62].

### 4.4. Anti-Inflammatory and Anti-Nociceptive Properties of Psophocarpus tetragonolobus

Winged bean seeds have a potential anti-inflammatory activity due to the presence of a peptide that can act as an inhibitor for angiotensin-converting enzyme [63]. Moreover, the anti-nociceptive and anti-inflammatory properties of six Malaysian medicinal plants were evaluated, including *Psophocarpus tetragonolobus*. The anti-inflammatory and anti-nociceptive activities of the plant extracts were evaluated by using Griess assay and by measuring the number of writhing response of mice upon acetic acid induction respectively. All plants showed significant nitric oxide (NO) inhibitory activity upon IFN-γ/LPS treated macrophages in a concentration-dependent way without causing cytotoxicity to RAW 264.7 cells; in addition to that all plants lead to the suppression of writhing response of mice at different degrees of inhibition (10.7 to 43.1%) at 0.2 g/kg [64].

## 5. Conclusions

Due to all the positive nutrition benefits offered by winged bean, *P. tetragonolobus* could be a replacement in various food formulations as a functional ingredient. Moreover, *P. tetragonolobus* has been commonly used in traditional medicine for many years. Winged bean possess bioactive phytochemicals with antioxidant properties, so it’s considered a putative promising resource for treating diseases related to oxidative stress and inflammatory reactions. Thus, this plant can serve as sources of health-promoting nutrients and phytochemicals for human and animals.

## Figures and Tables

**Table 1:** Plant taxonomy.

Subkingdom:	*Tracheobionta*
Super division:	*Spermatophyta*
Division:	*Mangoliophyta*
Class:	*Mangoliopsyda*
Subclass:	*Rosidea*
Order:	*Fabales*
Family:	*Fabaceae*
Subfamily:	*Papilionoideae*
Tribe:	*Phaseoleae*
Genus:	*Psophocarpus*
Species:	*P. tetragonolobus*

**Table 2:** Main metabolites responsible of the biological activity of *Psophocarpus tetragonolobus.*

Metabolites	Activity	Plant Part	Country	References
Phytate	Anti-nutritional factors, affinity for specific blood cell antigens	Seed	Malaysia	[36,42]
Tannin	Nonspecific enzymes inhibitors, hemagglutinin activity	Seed	Malaysia	[42]
Psophocarpin	Chemotrypsin inhibitory activity	Seed, pods	India	[44,45]
Lectin	Hemagglutinin activity	Mainly in seed, roots	India	[35,46,47]
Albumin 1 (WBA-1)	Kunitz-type trypsin inhibitors	Seed	Malaysia	[37]
Phaseolin	α-amylase inhibitor	Seed	Malaysia	[48]
